# A Case of Factor XIII Deficiency Identified by Recurrent Postoperative Bleeding After Tonsillectomy

**DOI:** 10.1002/ccr3.72006

**Published:** 2026-02-06

**Authors:** Takanobu Teramura, Akihiro Sakai, Masashi Hamada, Koichiro Wasano, Ai Yamamoto, Hikaru Yamamoto, Kenji Okami

**Affiliations:** ^1^ Department of Otolaryngology, Head and Neck Surgery Tokai University, School of Medicine Isehara Japan

**Keywords:** factor XIII deficiency, normal PT and APTT, occult coagulation disorder, postoperative bleeding, recurrent hemorrhage, tonsillectomy

## Abstract

Postoperative bleeding is a well‐known complication of tonsillectomy. Although inadequate hemostasis and vascular injury are common causes, occult coagulation disorders may also contribute. Factor XIII deficiency is an extremely rare condition that is particularly difficult to detect preoperatively because PT and APTT typically remain normal. A 27‐year‐old man underwent bilateral tonsillectomy for a recurrent peritonsillar abscess. Despite normal coagulation screening, the patient developed repeated postoperative hemorrhages requiring surgical management. Further evaluation revealed reduced Factor XIII activity (36%). After Factor XIII concentrate administration, bleeding ceased, and the postoperative course stabilized. Factor XIII deficiency should be considered in patients with recurrent postoperative bleeding despite normal routine coagulation tests. Early recognition and timely replacement therapy may prevent severe complications.

## Key Clinical Message

Postoperative bleeding is a known complication of tonsillectomy. The most common causes include inadequate hemostasis or vascular injury; rarely, coagulation disorders can also be implicated. Unlike hemophilia, which alters APTT, Factor XIII deficiency typically presents with normal PT and APTT levels, making preoperative detection more difficult.

## 1 Introduction

Postoperative bleeding is a common complication of tonsillectomy. The primary causes include inadequate intraoperative hemostasis or vascular injury, and postoperative vascular damage resulting from scab detachment or infection. However, cases in which postoperative hemorrhage led to the diagnosis of underlying coagulation disorders, such as hemophilia, have been reported [1, 2]. In such conditions, the activated partial thromboplastin time (APTT) is typically prolonged, allowing for the detection of these abnormalities during routine preoperative coagulation screening. When coagulation abnormalities are identified, appropriate factor replacement therapy can be administered before surgery to reduce the risk of bleeding. However, XIII coagulation factor deficiency is critical for final stages of the coagulation cascade and has been reported to cause no abnormalities in prothrombin time (PT) or APTT [3]. Congenital Factor XIII deficiency is a rare genetic bleeding disorder. In the United States, its incidence is estimated to be approximately 1 in 3–5 million live births. By contrast, acquired Factor XIII deficiency is uncommon and may result from autoimmune mechanisms or increased consumption associated with surgery, trauma, or systemic diseases. Because routine coagulation tests remain normal in both forms, the true prevalence is likely to be underestimated.

Here, we describe a case of recurrent postoperative bleeding after tonsillectomy that required hemostasis under general anesthesia. Subsequent coagulation factor testing confirmed reduced Factor XIII levels. After administration of Factor XIII concentrate, no further bleeding occurred, and the patient experienced an uneventful recovery. Here, we present this case along with a review of the relevant literature.

## 2 Case History/Examination

A 27‐year‐old male patient presented with recurrent episodes of fever and a sore throat. The patient's medical and family histories were unremarkable. The patient had no history of bleeding, delayed wound healing, or hematoma formation. He was a nonsmoker and occasionally consumed alcohol. To date, no drug allergies have been reported.

The patient had experienced four episodes of peritonsillar abscess (PTA) over the past several years (at X‐9, X‐7, X‐6, and X years before presentation). The patient was referred to our department for palatine tonsillectomy due to recurrent PTA. Physical examination revealed that the palatine tonsils were enlarged (Friedman grade II). Preoperative laboratory assessments, including biochemical and coagulation tests, were within normal limits (Table 1).

**TABLE 1:** Laboratory findings.

Test	Result
Hematology	
WBC	7000/μL
RBC	5.37 × 10^6^/μL
Hb	16.6 g/dL
PLT	260 × 10^3^/μL
Biochemistry	
AST	20 U/L
ALT	33 U/L
BUN	12 mg/dL
Cre	0.7 mg/dL
Glu	106 mg/dL
Coagulation tests	
APTT	33 s
PT	11.1 s
PT‐INR	0.97

## 3 Investigations and Treatment

### 3.1 Clinical Course

Bilateral tonsillectomy was performed on Y, Z, and X. Both palatine tonsils were excised extracapsular using bipolar cautery. Moderate pericapsular adhesions were observed, consistent with scarring from prior episodes of peritonsillar abscesses. The operative time was 25 min, with minimal intraoperative blood loss. The patient was discharged on postoperative day (POD) Z + 5 without immediate complications. However, on POD Z + 5, the patient experienced postoperative hemorrhage and was readmitted for hemostatic treatment under general anesthesia. On Z + 11, the patient presented with massive postoperative bleeding, necessitating a second hemostatic procedure under general anesthesia. Despite undergoing the usual surgery, the patient experienced multiple episodes of postoperative bleeding and poor granulation tissue formation at the wound site. These findings raised suspicion of an underlying coagulation disorder and prompted additional laboratory evaluations.

### 3.2 Coagulation Function Test

On POD Z + 12, coagulation tests revealed a 36% decrease in Factor XIII activity. PT was mildly prolonged, whereas APTT remained within normal limits. There was no decrease in the total plasminogen activator inhibitor‐1 (PAI‐1), protein C, or protein S activity, which are factors of the fibrinolytic system, or an increase in FDP or D‐dimer levels. Based on these results, Factor XIII deficiency was diagnosed (Table 2).

**TABLE 2:** Coagulation and protein activity findings.

Test	Result	Reference range
APTT	35 s	(25–36 s)
PT	14.2 s	(9.3–13.8 s)
PT‐INR	1.2	(0.8–1.10)
Blood Coagulation Factor VII Activity	66%	(75%–140%)
Blood Coagulation Factor VIII Activity	67%	(60%–150%)
Blood Coagulation Factor IX Activity	81%	(70%–130%)
von Willebrand Factor (vWF) Activity	103%	(60%–170%)
Blood Coagulation Factor XIII Activity	36%	(70%–140%)
Blood Coagulation Factor XIII Antigen	50%	(> 70%)
Total PAI‐1	41 ng/mL	(< 50%)
Protein C Activity	151%	(64%–146%)
Protein S Activity	94%	(67%–164%)
Protein S Antigen	92%	(73%–137%)
D‐dimer	0.5 μg/mL	(< 1.0)
Fibrin Degradation Products (FDP)	< 2.5 μg/mL	(< 5.0)

## 4 Outcome and Follow‐Up

### 4.1 Treatment Courses and Outcome

**FIGURE 1:**
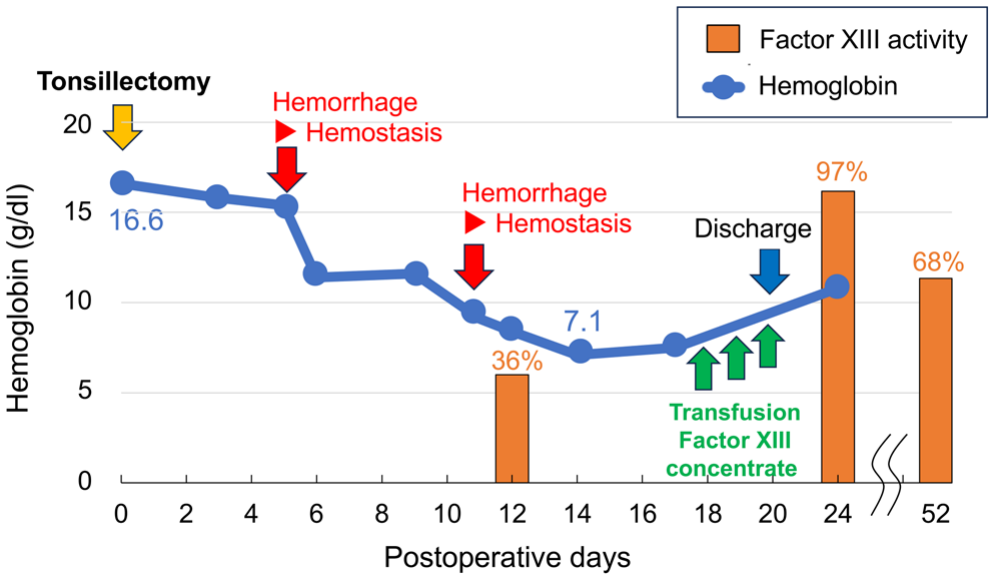
Postoperative course. Postoperative bleeding occurred on the 5th day after tonsillectomy and stopped under general anesthesia. Furthermore, on the 11th postoperative day, postoperative bleeding recurred, and hemostasis was performed under general anesthesia. Factor XIII activity measured on the 12th postoperative day was as low as 36%. Factor XIII blood transfusion was performed from the 18th to 20th postoperative days. Blood sampling on the 24th postoperative day showed that Factor XIII activity increased to 97%.

Following the second hemostatic intervention on POD Z + 12, the patient experienced no further bleeding episodes. Fibrogamin P (Factor XIII concentrate) was administered for 3 days from Z + 18 to Z + 20. The patient was subsequently discharged with Z + 20. At outpatient follow‐up, there were no recurrent bleeding events, and Factor XIII activity increased to 97% with Z + 24. Postoperative wounds showed normal mucosal healing. However, by Z + 52, the Factor XIII activity decreased to 68% in subsequent blood samples (Figure 1).

## 5 Discussion

Coagulation Factor XIII plays an important role in hemostasis and wound healing. It belongs to the transglutaminase family, a group of enzymes that crosslink proteins and function as fibrin‐stabilizing factors. Factor XIII circulates as an A2B2 heterotetramer comprising a catalytic A‐subunit dimer and a stabilizing B‐subunit dimer. During the final stage of the coagulation cascade, Factor XIII cross‐links fibrin strands, thereby strengthening and stabilizing the clot. Consequently, Factor XIII deficiency results in reduced clot stability and premature clot dissolution before adequate tissue repair, leading to persistent bleeding and delayed wound healing [4]. Because Factor XIII acts only at the terminal step of the coagulation pathway and is independent of the intrinsic and extrinsic pathway reactions, neither PT nor APTT is affected in cases of Factor XIII deficiency.

Factor XIII deficiency can be broadly classified into congenital and acquired forms. Congenital deficiency results from defects in either the A‐ or B‐subunit and is present at birth, although extremely rare. On the other hand, acquired Factor XIII deficiency is caused by anti‐factor XIII autoantibodies or inhibitors, or by increased consumption of Factor XIII in conditions such as surgery, trauma, malignancy, disseminated intravascular coagulation, sepsis, and liver failure [5, 6, 7]. In both congenital and acquired forms, routine coagulation parameters, including PT, APTT, platelet count, and platelet aggregation, are generally normal, making diagnosis challenging.

The diagnostic criteria for autoimmune Factor XIII deficiency (AHFXIII/13) are summarized in Table 3 [8]. In our case, the patient met criteria (1–5), fulfilling the definition of a possible diagnosis. However, as inhibitor and autoantibody testing were not performed, the diagnostic certainty could not be elevated beyond this level.

**TABLE 3:** Diagnostic criteria for autoimmune hemorrhaphilia FXIII/13.

Diagnostic category	Criteria
Possible	Consider AHFXIII/13 if all the following conditions are met:
	(1) Recent onset of bleeding symptoms mainly in the older adult.
	(2) No family history of congenital/inherited deficiency of FXIII or other coagulation factors.
	(3) Lack of previous bleeding symptoms especially in association with previous hemostatic challenges (e.g., surgery, invasive tests, trauma, etc.)
	(4) Not explained by excessive medication such as anticoagulants and antiplatelet drugs.
	(5) Abnormality of FXIII parameter(s) on laboratory testing (FXIII activity and/or antigen < 50%)
Probable	In addition to criteria (1) to (5) above:
	(6) Presence of FXIII inhibitors* (positive by cross‐mixing tests between patient's and healthy control's plasma using standard functional tests after 2 h incubation at 37°C)
Definite	In addition to criteria (1) to (5) above:
	(7) Presence of anti‐FXIII autoantibodies (positive by immunological methods)

*Note:* The asterisk (*) indicates a methodological note.

The mainstay treatment for Factor XIII deficiency is replacement therapy with highly purified plasma‐derived Factor XIII concentrates (e.g., Corifact/fibrogammin P) or recombinant Factor XIII‐A2 (catridecacog) [9]. In this case, 720 to 1440 units of Fibrogammin P were administered daily for 5 days according to the product guidelines. Although freshly frozen plasma (FFP) contains Factor XIII, its low concentration necessitates large infusion volumes, which increases the risk of volume overload. Therefore, concentrated preparations are preferred for replenishment.

In our patient, both the Factor XIII activity and antigen levels were reduced. Despite successful bleeding control following replacement therapy, Factor XIII activity remained low on follow‐up testing, suggesting possible acquired Factor XIII deficiency due to autoantibodies or inhibitors. In addition, substantial postoperative blood loss (totaling approximately 2300 mL) may have contributed to the increased consumption, further supporting a secondary acquired deficiency.

Associations between recurrent post‐tonsillectomy bleeding and reduced Factor XIII activity have been reported by Albahkaly et al. [3], Windfuhr et al. [10], Jankovic et al. [5], and Altamimi et al. [11]. Across these cases, patients required transfusions of Factor XIII concentrate, FFP, or red blood cells. The reported ages ranged from 4 to 40 years and the number of rebleeding episodes ranged from 2 to 7 years. Factor XIII activity at the time of bleeding ranges from 7% to 58%. Notably, the case described by Albahkaly et al. involved a 4‐year‐old girl who died.

Most of the affected patients demonstrated normal preoperative coagulation profiles [5, 10, 11]. For individuals with a personal or family history suggestive of a bleeding disorder, or when the family history is unknown, preoperative evaluation of specific coagulation factors may be warranted [11]. In addition, clinicians should consider Factor XIII deficiency when postoperative bleeding recurs despite normal coagulation screening, particularly when bleeding is delayed or wound healing is impaired. This suspicion is especially important in adults with no history of bleeding because acquired Factor XIII deficiency often presents for the first time after surgery or trauma. When preoperative Factor XIII activity is < 60%, prophylactic supplementation is recommended to reduce surgical bleeding risk [12].

The long‐term management of congenital Factor XIII deficiency requires regular prophylactic replacement therapy, which significantly reduces the risk of life‐threatening bleeding, including intracranial hemorrhage. In contrast, acquired Factor XIII deficiency has a highly variable prognosis, depending on the underlying cause. Autoimmune forms are associated with high morbidity and mortality and often require immunosuppressive therapy in addition to factor replacement. Because Factor XIII activity may fluctuate over time, periodic monitoring is recommended, even after apparent stabilization [9].

In the present case, reduced Factor XIII activity was identified only after repeated postoperative hemorrhage and was successfully corrected with supplementation. In patients with persistent or recurrent postoperative bleeding, additional evaluation of the coagulation–fibrinolytic system, including Factor XIII activity, anti‐factor XIII antibodies, Factor XIII inhibitors, FDP, and D‐dimer levels, should be performed. On the basis of these findings, timely supplementation should be considered to prevent further complications.

## 6 Conclusion

In summary, we reported a case of reduced Factor XIII activity in a patient who developed multiple episodes of postoperative hemorrhage after tonsillectomy. Following Factor XIII replacement therapy, the patient had an uneventful course with no further bleeding. This case highlights the possibility that coagulation disorders that are undetectable on routine preoperative screening may still be present. Therefore, when patients experience recurrent postoperative bleeding or repeated complications, it is clinically important to perform additional diagnostic evaluations to identify the potential underlying coagulation abnormalities.

In particular, clinicians should consider evaluating Factor XIII activity when postoperative bleeding recurs despite normal PT and APTT, when bleeding is delayed during the postoperative course, or when wound healing is impaired. Early recognition of Factor XIII deficiency and timely administration of replacement therapy can prevent severe complications and should be incorporated into the differential diagnosis of unexplained postoperative hemorrhage in otolaryngological practice.

## 7 Limitation

This study has several limitations. First, because inhibitor assays and anti‐factor XIII autoantibody tests were not performed, we were unable to determine whether the reduced Factor XIII activity represented a congenital deficiency, an acquired form, such as autoimmune Factor XIII deficiency, or consumption secondary to surgery. Second, this study describes a single case, which limits the generalizability of the findings. Larger case series and systematic evaluations are required to clarify the clinical characteristics, diagnostic approaches, and optimal management of postoperative bleeding associated with Factor XIII deficiency.

## Author Contributions

**Takanobu Teramura:** conceptualization, investigation, methodology, writing – original draft, writing – review and editing. **Akihiro Sakai:** investigation, supervision, writing – review and editing. **Masashi Hamada:** investigation, methodology. **Koichiro Wasano:** investigation, methodology. **Ai Yamamoto:** investigation. **Hikaru Yamamoto:** supervision. **Kenji Okami:** supervision.

## Funding

The authors have nothing to report.

## Ethics Statement

All protected health information was withheld from this piece to ensure patient anonymity.

## Consent

We have obtained written informed consent from the participant presented in this report.

## Conflicts of Interest

The authors declare no conflicts of interest.

## Data Availability

All relevant data has been presented in this manuscript.
